# *T. gondii* excretory proteins promote the osteogenic differentiation of human bone mesenchymal stem cells via the BMP/Smad signaling pathway

**DOI:** 10.1186/s13018-024-04839-0

**Published:** 2024-07-01

**Authors:** Mingzhu Deng, Feifei Gao, Tianfeng Liu, Weiqiang Zhan, Juanhua Quan, Ziquan Zhao, Xuyang Wu, Zhuolan Zhong, Hong Zheng, Jiaqi Chu

**Affiliations:** 1 Orthopaedic Center, Affiliated Hospital of Guangdong Medical University, Zhanjiang, China; 2 Stem Cell Research and Cellular Therapy Center, Affiliated Hospital of Guangdong Medical University, Zhanjiang, China; 3 Laboratory of Gastroenterology, Affiliated Hospital of Guangdong Medical University, Zhanjiang, China

**Keywords:** Bone marrow mesenchymal stem cells, *Toxoplasma gondii*, Excretory proteins, Osteogenic differentiation, BMP/Smad signaling pathway, Bone defect

## Abstract

**Background:**

Bone defects, resulting from substantial bone loss that exceeds the natural self-healing capacity, pose significant challenges to current therapeutic approaches due to various limitations. In the quest for alternative therapeutic strategies, bone tissue engineering has emerged as a promising avenue. Notably, excretory proteins from *Toxoplasma gondii* (TgEP), recognized for their immunogenicity and broad spectrum of biological activities secreted or excreted during the parasite’s lifecycle, have been identified as potential facilitators of osteogenic differentiation in human bone marrow mesenchymal stem cells (hBMSCs). Building on our previous findings that TgEP can enhance osteogenic differentiation, this study investigated the molecular mechanisms underlying this effect and assessed its therapeutic potential in vivo.

**Methods:**

We determined the optimum concentration of TgEP through cell cytotoxicity and cell proliferation assays. Subsequently, hBMSCs were treated with the appropriate concentration of TgEP. We assessed osteogenic protein markers, including alkaline phosphatase (ALP), Runx2, and Osx, as well as components of the BMP/Smad signaling pathway using quantitative real-time PCR (qRT-PCR), siRNA interference of hBMSCs, Western blot analysis, and other methods. Furthermore, we created a bone defect model in Sprague-Dawley (SD) male rats and filled the defect areas with the GelMa hydrogel, with or without TgEP. Microcomputed tomography (micro-CT) was employed to analyze the bone parameters of defect sites. H&E, Masson and immunohistochemical staining were used to assess the repair conditions of the defect area.

**Results:**

Our results indicate that TgEP promotes the expression of key osteogenic markers, including ALP, Runx2, and Osx, as well as the activation of Smad1, BMP2, and phosphorylated Smad1/5—crucial elements of the BMP/Smad signaling pathway. Furthermore, in vivo experiments using a bone defect model in rats demonstrated that TgEP markedly promoted bone defect repair.

**Conclusion:**

Our results provide compelling evidence that TgEP facilitates hBMSC osteogenic differentiation through the BMP/Smad signaling pathway, highlighting its potential as a therapeutic approach for bone tissue engineering for bone defect healing.

**Supplementary Information:**

The online version contains supplementary material available at 10.1186/s13018-024-04839-0.

## Introduction

Bone defects pose a common challenge in orthopedic diseases, characterized by a large volume of bone loss that exceeds the body’s natural capacity for self-repair [1]. Various causes contribute to bone defects, including severe trauma, infection, tumor resection, and other injuries [2]. These conditions not only impair an individual’s quality of life but also result in substantial healthcare costs and economic burdens on individuals, families, societies, and health-care systems. Although several methods exist for addressing bone defects, their effectiveness remains controversial [3]. Current approaches for treating bone defects include bone grafts, distraction osteogenesis, the induced membrane technique, free fibula vascularized grafts and the use of biomaterials [4]. Although these techniques may be largely beneficial, none have been proven to be completely satisfactory [5]. Thus, it is critical to develop new techniques and strategies for the treatment of bone defects [6].

Bone marrow mesenchymal stem cells (BMSCs), which reside in bone marrow, exhibit self-renewal and multilineage differentiation capabilities, enabling them to differentiate into osteoblasts, adipocytes, endotheliocytes, and chondrocytes [7]. BMSCs are characterized by advantageous features for regenerative therapy, such as high proliferation potential, multilineage differentiation capability, anti-inflammatory and immune-modulatory properties, support for hematopoiesis, and stimulation of angiogenesis [8–10]. Consequently, researchers have explored the use of BMSCs, including human bone marrow mesenchymal stem cells (hBMSCs), as seed cells in bone tissue engineering for bone defect repair [11]. Bone tissue engineering leverages seed cells [12], cell growth factors [13], and biomimetic materials as bone substitutes [14, 15] and provides a template for guiding bone regeneration and achieving the goal of bone defect healing [16].

*Toxoplasma gondii* (*T. gondii*, Tg) is a protozoan parasite capable of infecting both humans and animals. It has three stages of infection: tachyzoite, bradyzoite and sporozoite infection [17]. During its invasion and replication within host cells, *T. gondii* excretes and secretes numerous proteins and other molecules, which are collectively known as excretory proteins (EPs) [18–20]. Studies have shown that many of these constituents, such as microneme proteins (MICs), dense granule proteins (GRAs), rhoptry proteins (ROPs) and cytoskeleton proteins, induce immune responses [21, 22]. Furthermore, these excretory proteins are believed to play essential roles in *T. gondii* reproduction, localization, invasion of host cells and regulation of the host immune response [23, 24]. Some studies have explored the potential applications of TgEP, such as its use as an antigen in DNA vaccines and for improving the sensitivity and specificity of toxoplasmosis diagnosis [25]. Recent research conducted by our team has demonstrated that TgEP significantly enhances osteogenic differentiation in BMSCs via the modulation of aerobic glycolysis [26].

Bone morphogenetic proteins (BMPs) are a group of signaling molecules that belong to the transforming growth factor-beta (TGF-β) superfamily. BMPs play a crucial role in regulating the differentiation of mesenchymal stem cells (MSCs) and the process of bone formation [27]. BMPs, including BMP-2, BMP-4, BMP-6, and BMP-9, have been shown to induce the osteogenic differentiation of MSCs both in vitro and in vivo [28]. BMP-2 is essential for postnatal skeletal homeostasis and plays a crucial role in innate bone repair [29]. Upon activation and transphosphorylation, BMP receptors recruit and phosphorylate receptor-regulated Smads (R-Smads), such as Smad1/5/8 [30]. Subsequent phospho-Smad1/5/8 binding with Smad4 results in the formation of a heterodimeric complex that translocates to the nucleus, where it stimulates or inhibits the expression of target genes [31]. Moreover, in the nucleus, the BMP/Smad signaling pathway activates the transcription factor Runx2, which initiates a cascade of downstream genes involved in osteogenesis [32].

In the present study, the primary objective was to further verify the impact of TgEP on the osteogenic differentiation of BMSCs, as well as its potential involvement in the BMP/Smad signaling pathway. Additionally, by conducting in vivo experiments to examine the effects of TgEP on bone defect repair, this study may contribute to advancements in regenerative medicine and provide useful references for future research and clinical applications in orthopedics.

## Materials and methods

### Isolation and culture of hBMSCs

Primary human BMSCs (hBMSCs) were isolated from the bone marrow aspirates of patients who required hip replacement due to aseptic necrosis of the femoral head. Informed consent was obtained from all participants before their inclusion in the research. hBMSCs were isolated by density gradient centrifugation using Ficoll-Histopaque-1077 (Sigma‒Aldrich, USA) [33]. The isolated cells were then cultured in MEM-Alpha medium supplemented with 10% fetal bovine serum (FBS) and 1% penicillin and streptomycin. The culture medium was changed every 3 days until the cells became confluent. Cells from passages 4 to 6 (P4−6) were used in subsequent experiments. The present study was approved by the Medical Ethical Committee of the Affiliated Hospital of Guangdong Medical University (approval number PJKT2023-024).

### Phenotypic characterization of hBMSCs

To characterize the phenotype of the cells, P4 hBMSCs were incubated with fluorescein-conjugated antibodies targeting CD90 (BD Biosciences, USA), CD73 (BD Biosciences), CD105 (BD Biosciences), CD19 (BioLegend, USA), and CD11b (Invitrogen, USA) for 30 min at room temperature. Flow cytometry analysis was conducted using a BD FACSVia™ flow cytometer (Becton Dickinson, USA), and the data were analyzed with FlowJo software (vX0.7).

### *T. gondii* maintenance and TgEP extraction

The RH strain (type I) of *T. gondii* was kindly provided by Dr. Young‑Ha Lee, Department of Infection Biology, Chungnam National University School of Medicine, Republic of Korea, and the method of TgEP extraction was slightly modified according to previous work [34, 35]. Briefly, tachyzoites of the RH strain *T. gondii* were revived and maintained via intraperitoneal passages in C57BL/6 mice and subsequently proliferated in vitro through the human retinal pigment epithelial cell line ARPE-19 (American Type Culture Collection, USA). Then, sufficient tachyzoites were collected and washed twice with PBS, and 0.5 ml of Hank’s balanced salt solution was added for every 3 × 10^8^ tachyzoites. The suspension of the *T. gondii* RH strain was incubated in a constant temperature shaker (37 °C, 3.57 g) for 4 h. The resulting suspension was centrifuged three times at 14,000×g at 4 °C for 3 min, after which the supernatant was collected, which was the desired TgEP. The protein concentration of TgEP was determined using an Enhanced BCA Protein Reagent kit (Beyotime, China).

### Induction of hBMSC osteogenic, chondrogenic and adipogenic differentiation

Osteogenic differentiation of hBMSCs was induced using osteogenic induction medium (OIM). The OIM components included low-glucose Dulbecco’s modified Eagle’s medium (L-DMEM), 100 nM dexamethasone, 100 IU/mL penicillin/streptomycin, 10% fetal bovine serum (FBS, v/v), 10 mM β-glycerophosphate, and 50 µM L-ascorbate-2-phosphate (Sigma‒Aldrich, USA). For chondrogenic differentiation, the cells were incubated in chondrogenic induction medium, which was based on MEM-Alpha Medium enriched with 20 nM dexamethasone, 100 IU/mL penicillin/streptomycin, 1% FBS (v/v), 1× insulin-transferrin-selenium (ITS), 5 µg/mL L-ascorbate-2-phosphate (Sigma‒Aldrich, USA), and 10 ng/mL transforming growth factor-beta1 (TGF-β1; PeproTech, USA). Adipogenic differentiation was induced using lipogenesis induction medium composed of high-glucose Dulbecco’s modified Eagle’s medium (H-DMEM) supplemented with 500 nM dexamethasone, 100 IU/mL penicillin/streptomycin, 10% FBS (v/v), 50 µM indomethacin, 0.5 mM isobutylmethylxanthine, and 10 ng/mL insulin (Sigma‒Aldrich, USA).

Upon reaching 80% confluency, the cells were segregated into different groups for treatment. The culture medium was then replaced with fresh OIM, chondrogenic, or lipogenesis induction medium, each containing varying concentrations of TgEP (0, 1, or 10 µg/mL) for predetermined durations. The maintenance of the cells involved refreshing the respective induction medium every three days.

### Cell cytotoxicity and cell proliferation assays

The hBMSCs were seeded into a 96-well plate at a density of 3000 cells per well and allowed to adhere for 12 h. To evaluate the cytotoxicity of TgEP, a CytoTox 96 nonradioactive cytotoxicity assay (Promega, USA) was performed. To assess cell proliferation, a Cell Counting Kit-8 (Beyotime, China) was used. Cells were treated with various concentrations of TgEP (0, 1, 2, 10, or 50 µg/mL) for 24 h.

### Alkaline phosphatase staining, Alizarin red S staining and oil red O staining

hBMSCs were seeded in a 12-well plate at a density of 6 × 10^4^ cells per well. Once the cells adhered to the plate, the medium was replaced with OIM or adipogenic or chondrogenic induction medium. ALP staining solution (LEAGENE^®^, China) and alizarin red or oil red O (OriCell^®^, China) were used to determine the ALP content and the formation of mineralized nodules and transparent oil droplet-like substances, respectively.

### Alcian blue staining

A total of 5 × 10^5^ hBMSCs were resuspended in chondrogenic induction medium, and the cell clusters were allowed to settle in a 1.5 mL conical-bottomed polypropylene test tube. The medium was replaced every 3 days for a total of 14 days to facilitate the formation of cartilage tissue blocks. The tissue blocks were sectioned and subsequently stained with Alcian blue solution (OriCell^®^, China) to visualize the presence of positive cells that particularly produced acidic polysaccharides.

### Quantitative real-time PCR (qRT‒PCR)

Total RNA was isolated from hBMSCs using NucleoZOL (MACHEREY-NAGEL, Germany). The concentration and purity of RNA were determined by measuring the absorbance at 260 nm using a NanoDrop ONEC spectrophotometer (Thermo Fisher Scientific, USA). Subsequently, the RNA was reverse transcribed into cDNA using the HiScript™ III RT SuperMix for qPCR kit (Vazyme, China) following the manufacturer’s instructions.

The ABI 7500 Real-Time PCR System (Thermo Fisher Scientific, USA) was used for qRT‒PCR. ChamQ Universal SYBR qPCR Master Mix (Vazyme, China) was used for qRT‒PCR. The relative expression levels of the target genes were calculated using the 2^−△△Ct^ method and normalized to those of internal controls. The forward and reverse primers used for amplification of each target gene are shown in Table S1.

### siRNA interference of hBMSCs

hBMSCs were transfected with BMP2 siRNA (siBMP2) (GenePharma, China) using Lipofectamine™ RNAiMAX (Thermo Fisher, USA). The transfection was carried out for 24 h, and then the medium was replaced with either regular cell growth medium or OIM. At specific time points, cell samples were collected for further analysis and subsequent experiments.

### Inhibitor treatment of hBMSCs

hBMSCs were treated with LDN193189 (Beyotime, China), a specific inhibitor of the BMP/Smad pathway. The hBMSCs were divided into four groups: the control group (treated with OIM only), the LDN group (treated with OIM and 5 µM LDN193189 inhibitor), the TgEP group (treated with OIM and 10 µg/mL TgEP) and the LDN + TgEP group (treated with OIM, 5 µM LDN193189 inhibitor and 10 µg/mL TgEP). To ensure efficient inhibition of the BMP/Smad pathway, the cells were pretreated with LDN193189 for 1 h, followed by different treatments according to the respective groups.

### Western blot analysis

Cells were lysed in RIPA lysis buffer (Thermo Fisher Scientific, USA), and the protein concentration was determined using an Enhanced BCA Protein Reagent kit (Beyotime, China). Approximately 20 µg of total protein was separated by 10% or 12% SDS‒PAGE and transferred onto polyvinylidene fluoride (PVDF) membranes (Millipore, USA). The PVDF membranes were then blocked with 5% (w/v) nonfat milk for 1 h at room temperature and incubated overnight at 4 °C with primary antibodies specific for β-actin (sc-47,778) (1:1000, Santa Cruz, USA), BMP2 (ab14933) (1:1000, Abcam, UK), ALP (Cat.#: R23427) (1:1000, ZEN-BIO, China), Runx2 (Cat.#: 860,139) (1:1000, ZEN-BIO, China), osteocalcin (OCN) (Cat.#: 514,636) (1:1000, ZEN-BIO, China), and Osterix (Osx) (ab22552) (1:1000, Abcam, UK). After rinsing, the membranes were incubated with HRP-labeled secondary antibodies for 1 h at room temperature. The proteins of interest were visualized using WesternLumaxLight™ Superior (ZETA Life, USA), and β-actin was utilized as the reference protein to normalize the protein expression levels.

### Immunofluorescence (IF) staining

The hBMSCs were cultured on glass coverslips placed in 12-well plates for 24 h. Following the induction of osteogenesis and treatment with TgEP (0, 1, or 10 µg/mL) for 3 days, the cells were rinsed and fixed with 4% formaldehyde for 15 min at room temperature. Subsequently, the cells were permeabilized with 0.1% Triton X for 30 min and blocked with 1% bovine serum albumin (BSA) for 30 min. Primary antibodies, such as anti-Runx2 (ab76956) (1:200, Abcam, UK) or anti-BMP2 (ab14933) (1:200, Abcam, UK), were added to the cells and incubated overnight at 4 °C. After washing, the cells were incubated with a secondary antibody (A11004) (1:500, Invitrogen; Thermo Fisher Scientific, USA) for 1 h. To visualize the cell nuclei, 4’,6-diamino-2-phenylindole (DAPI) staining (Vector Laboratories, USA) was performed for 5 min. Finally, the samples were examined using a laser confocal scanning microscope (Olympus, Japan), and images were captured using image management software.

### Immunocytochemistry (ICC) staining

For the ICC staining experiment, the cells were fixed with 4% formaldehyde and then incubated with 0.1% Triton X, 3% H_2_O_2_ solution, and 1% BSA for 30 min each. Subsequently, the cells were incubated with a primary anti-BMP2 antibody (ab14933) (1:500, Abcam, UK), followed by detection using 3,3’-diaminobenzidine (DAB) (MXB^®^biotechnologies, China) and hematoxylin (Solarbio, China). The cells were observed under a microscope, and images were captured using image management software.

### Animal model

To evaluate the osteogenic potential of TgEP, a bone defect model was established in 8-week-old Sprague‒Dawley (SD) male rats weighing approximately 200 g. The distal femur was selected as the site for creating the bone defect. GelMa hydrogel (Engineering for Life, China) was used as the scaffold material to fill the defects. The SD rats were randomly divided into four groups, with ten rats in each group. Sample collection was planned at two time points: 6 weeks and 8 weeks after the surgical procedure. Five rats were collected from each group at each time point. The experimental groups included the Sham group (sham operation without drilling), the Model group (bone defect created by drilling), the Model + Gel group (bone defect filled with Gel hydrogel), and the Model + Gel + TgEP group (bone defect filled with a mixture of Gel hydrogel and TgEP at a concentration of 10 µg/mL). The rats were provided normal food and water ad libitum under specific pathogen-free (SPF) conditions. All animal experimental protocols were approved by the Ethics Committee of the Experimental Animal Center of the Affiliated Hospital of Guangdong Medical University. Using a microdrill, a lateral bone defect 4 mm in diameter was created in the distal femur under anesthesia. The construction of 4 mm bone defect animal model can avoid penetrating the rat femur or drilling through the side of the femur, and we referred to previous literature to make aperture bigger than 3 mm diameter in critical size [36, 37]. The defect was then filled with the designated materials according to the experimental group. At 6 weeks or 8 weeks postoperation, femur specimens were collected, fixed in 4% paraformaldehyde for 72 h, and subsequently stored in 75% ethanol at room temperature for further processing.

### Osteogenesis of the GelMa hydrogel and TgEP

To evaluate the osteogenesis of the GelMa hydrogel and TgEP in vitro, the GelMa hydrogel was mixed with 10 µg/mL TgEP and cocultured with hBMSCs in a 12-well Transwell plate (Tissue Culture Plate Insert, LABSELECT^®^, China) for 7 days, and the results were compared with those of the GelMa hydrogel group. The cells were placed on the plate bottom, and 30 µL of the mixture was added to the Transwell plate, and the OIM was changed every 3 days. Alkaline phosphatase staining and alizarin red S staining were used to detect the osteogenic effect in hBMSCs.

### Measurement of IL-1β, IL-10 and IL-23 in rat serum

To evaluate antigenicity in the rat model, 15 male SD rats of the same age and weight were utilized for experiments measuring the concentrations of IL-1β (Cat.#: MM-0047R1), IL-10 (Cat.#: MM-0195R1), and IL-23 (Cat.#: MM-00414R1). Blood was collected from the rats using the orbital venous blood collection technique. Given that the blood collection window spanned 1 week, samples were taken one day prior to surgery (*n* = 5, referred to as the Sham group), and on days 1 and 7 after surgery (*n* = 10, with 5 rats each in the Model + Gel group and the Model + Gel + TgEP group). Serum was then separated from the blood and the concentrations of IL-1β, IL-10 and IL-23 were determined using ELISA kits (Jiangsu Meimian Industrial Co., Ltd, China) according to the manufacturer’s instructions. Rat heart, liver, spleen, lung and kidney tissues were harvested from rats in the Sham and Model + Gel + TgEP groups and subjected to H&E staining.

### Microcomputed tomography (micro-CT)

To assess bone formation at the femur bone defect sites, femur bone samples (*n* = 5 for each group) were subjected to micro-CT scanning at 6 and 8 weeks postsurgery. The scans were performed using a micro-CT imaging system (Scanco Medical, vivaCT 80, Switzerland) with X-ray energy settings of 70 kV and 114 µA and a slice thickness of 15.6 μm. Various parameters, including the bone volume fraction (BV/TV), bone mineral density (BMD), trabecular thickness (Tb.Th), trabecular number (Tb.N), and trabecular separation (Tb.Sp), were calculated based on the micro-CT images. Additionally, three-dimensional (3D) reconstructions of the bone samples were generated using CTvox.3.3 software to provide comprehensive visualization of the bone structure and formation.

### Histology and immunohistochemistry

Following micro-CT scanning, specimens from the rats (*n* = 5 for each group) were subjected to decalcification in a 12% EDTA-2Na solution (pH 7.2–7.4) at room temperature for a period of 6 weeks, and the solution was changed every 3 days. Subsequently, the decalcified specimens were embedded in paraffin, and 5 μm thick tissue sections were cut perpendicular to the long axis of the femur. These tissue sections were then deparaffinized and subjected to staining with hematoxylin and eosin (H&E) (Solarbio, China) and Masson trichrome staining kits (Solarbio, China) to visualize the newly formed bone and bone-like tissue. For immunohistochemistry analysis, tissue sections were stained according to standard protocols using anti-osteocalcin (OCN) antibodies (Cat.#: 614,487) (1:100, ZEN-BIO, China). All sections were dehydrated and examined using an upright Olympus microscope (BX53, Olympus, Japan).

### Statistical analysis

The data were analyzed using GraphPad Prism 8.0.1 software (GraphPad, USA). The results are presented as the mean ± standard deviation (SD). Statistical comparisons between two groups were performed using Student’s *t* test. For comparisons involving more than two groups, ordinary one-way ANOVA followed by Tukey’s multiple comparison test was utilized. A p value less than 0.05 was considered to indicate statistical significance.

## Results

### Characterization and identification of hBMSCs

P4−6 hBMSCs were utilized for the experiments. The P2 and P4 generations of hBMSCs reached 90% confluency on the 3rd day of cell growth, displaying a characteristic long spindle-shaped morphology (Fig. S1A), which is consistent with previous relevant studies [38, 39]. Subsequently, we subjected hBMSCs to osteogenic, lipogenic and chondrogenic induction media. The results demonstrated that the cells underwent osteogenic differentiation, the formation of mineralized nodules (red); lipogenic differentiation, the presence of transparent oil droplet-like substances (red); and chondrogenic differentiation, the production of acidic mucopolysaccharides (blue) (Figure S1B.a, b, c). Additionally, flow cytometry analysis of P4 hBMSCs revealed that 99% of the cells were positive for CD73, CD90 and CD105, confirming their mesenchymal stem cell phenotype. Only 0.03% of the cells were negative for CD19 and CD11b, indicating the absence of unwanted cell types (Fig. S1C).

### Impact of TgEP on the viability and proliferation of hBMSCs

**Fig. 1:**
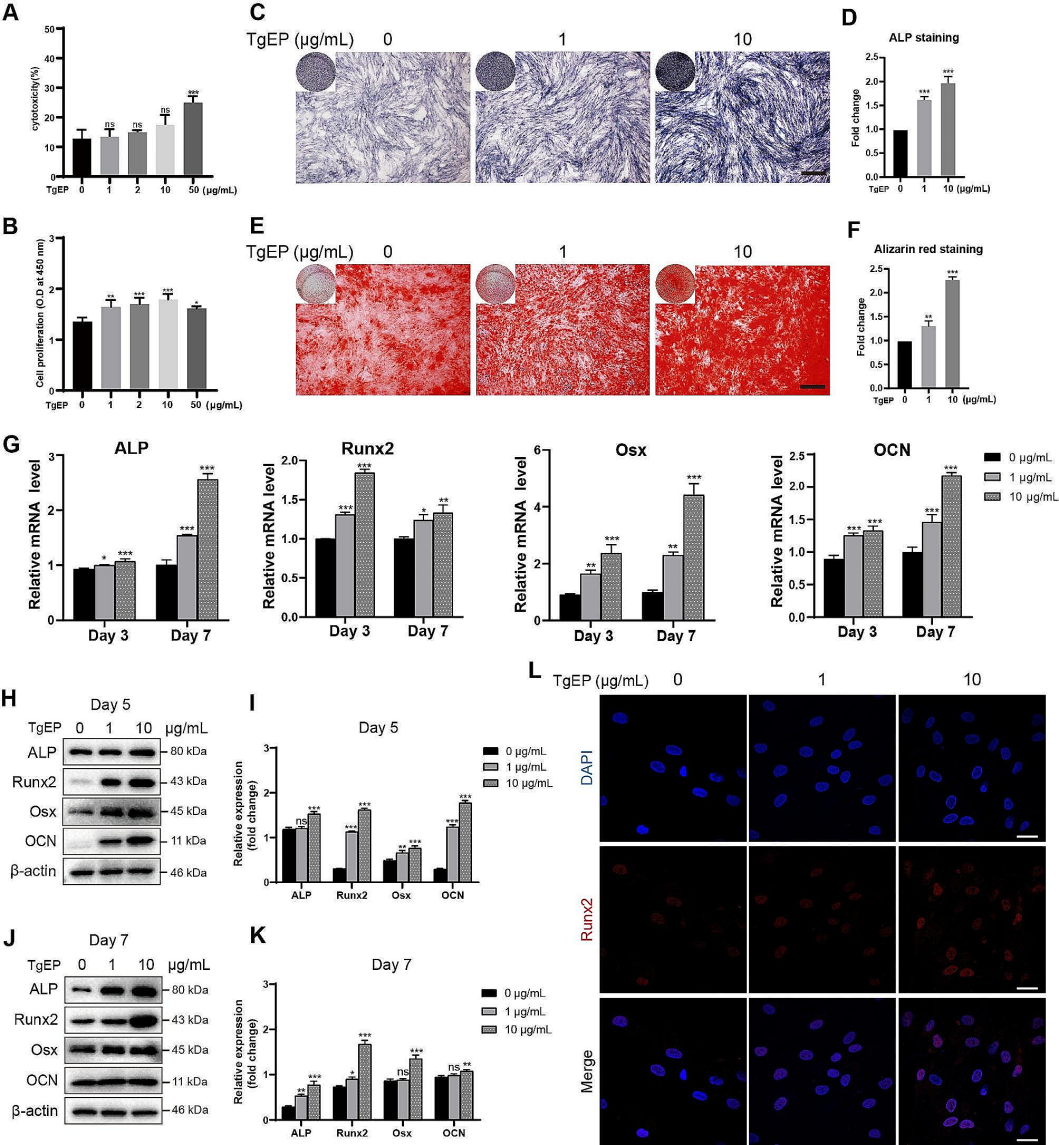
TgEP promoted the osteogenic differentiation of hBMSCs. (**A**) Cytotoxic effect of TgEP on hBMSCs detected using a cytotoxicity detection kit. (**B**) Effect of TgEP on cell proliferation assessed using a CCK-8 kit. (**C**) ALP staining was performed to evaluate ALP expression in the cells. Scale bar = 500 μm. (**D**) Quantification of ALP staining results using ImageJ software and statistical analysis. (**E**) Alizarin red staining was performed to measure the content of mineralized nodules in the cells. Scale bar = 500 μm. (**F**) Quantification of alizarin red staining results using ImageJ software and statistical analysis. (**G**) mRNA transcription levels of osteogenic genes were assessed using qRT‒PCR after the cells were treated for 3 or 7 days. (**H**, **J**) Western blotting analysis of the osteogenic proteins ALP, Runx2, Osx, and OCN in hBMSCs treated for 5 days (**H**) or 7 days (**J**). The expression levels were quantified using β-actin as a loading control (**I**, **K**). (**L**) Immunofluorescence staining was performed to detect the expression of Runx2 in the cells. Scale bar = 30 μm. (Data are presented as the mean ± SD; *n* = 3; ^*^P < 0.05, ^**^P < 0.01, ^***^P < 0.001, ^ns^*P*>0.05 compared to the control group)

Cytotoxicity analysis revealed that TgEP at concentrations ranging from 0 to 10 µg/mL did not exert any significant cytotoxic effect on hBMSCs. However, at a concentration of 50 µg/mL, TgEP exhibited noticeable cytotoxicity (Fig. 1A). Furthermore, the results of the CCK-8 assay demonstrated that TgEP concentrations between 0 and 10 µg/mL promoted cell proliferation. However, at a concentration of 50 µg/mL, TgEP inhibited cell proliferation (Fig. 1B). Therefore, subsequent experiments were performed using TgEP at concentrations of 1 and 10 µg/mL, and 0 µg/mL TgEP served as the negative control.

### TgEP promoted the osteogenic differentiation of hBMSCs

To explore the impact of TgEP on the osteogenic differentiation of hBMSCs, cells were cultured in OIM supplemented with varying concentrations of TgEP (0, 1, or 10 µg/mL) for certain days. After 5 or 7 days of treatment in each group, TgEP treatment led to enhanced ALP expression and elevated formation of mineralized nodules in hBMSCs (Fig. 1C, D, E, F). Moreover, qRT‒PCR analysis revealed that TgEP significantly upregulated the expression of ALP, Runx2, Osx, and OCN at 3 and 7 days, especially at a concentration of 10 µg/mL (Fig. 1G). Additionally, western blotting confirmed that TgEP treatment led to significant increases in the protein expression levels of Runx2, ALP, OCN, and Osx (Fig. 1H, I, J, K). To gain further insight into the underlying mechanism, we performed immunofluorescence staining after 3 days of osteogenic induction to assess the expression of the Runx2 protein. Notably, TgEP treatment increasingly induced the translocation of Runx2 (red) to the nucleus (blue) (Fig. 1L). These findings collectively demonstrate that TgEP enhances the osteogenic differentiation of hBMSCs in a concentration-dependent manner.

### TgEP regulated the osteogenic differentiation of hBMSCs via the BMP/Smad pathway

**Fig. 2:**
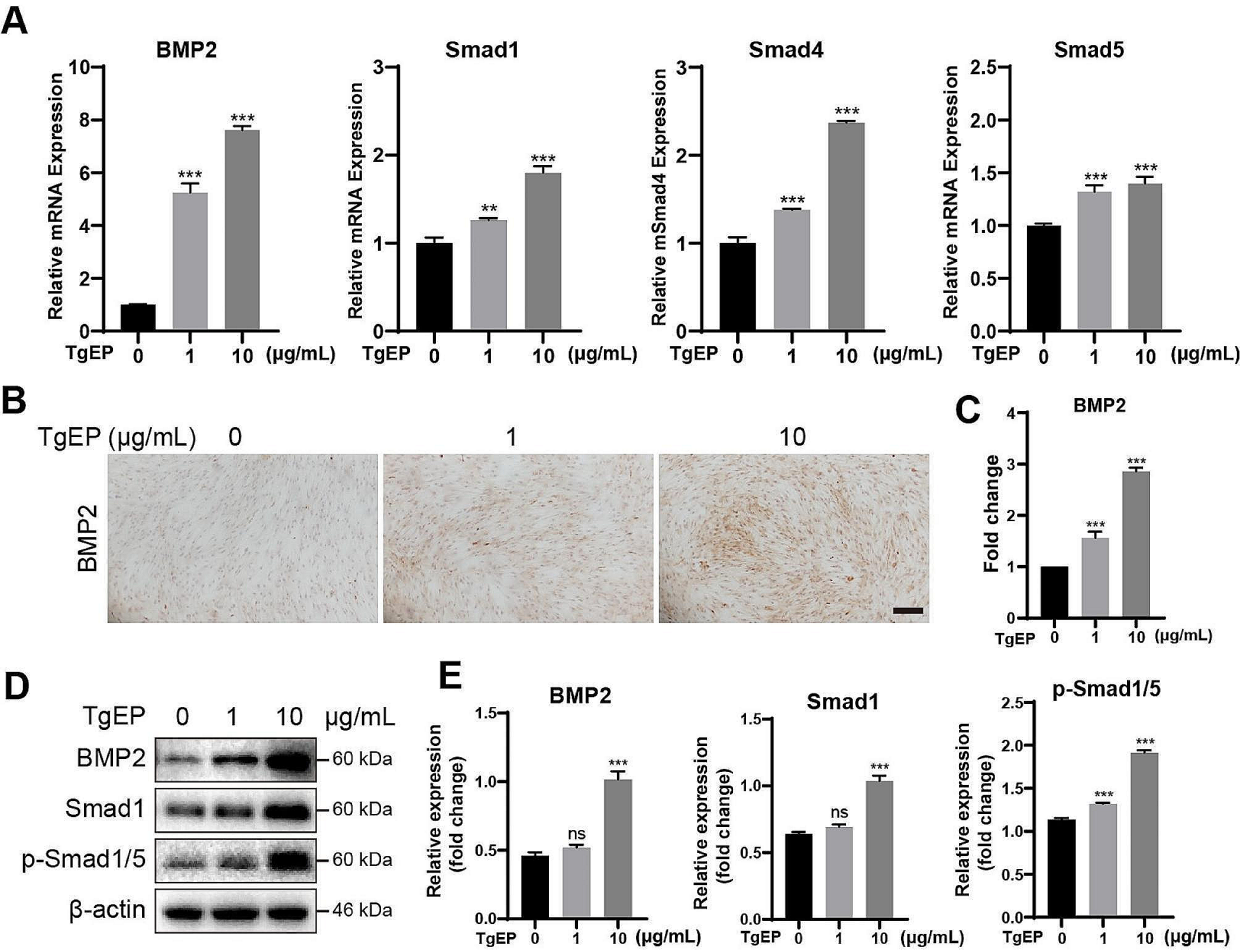
TgEP promoted BMP/Smad signaling pathway activation. (**A**) qRT‒PCR analysis of the transcription levels of BMP/Smad pathway-related genes after 3 days of treatment. (**B**) Immunocytochemical detection of BMP2 expression in cells after 3 days of treatment. (**C**) Quantification of immunocytochemistry results using ImageJ software and statistical analysis. (**D**) Western blotting analysis of BMP/Smad pathway-related proteins in cells after 7 days of treatment, with quantification of protein expression normalized to that of β-actin (**E**). (The data are presented as the means ± SDs; *n* = 3; ^**^P < 0.01, ^***^P < 0.001, ^ns^*P*>0.05 compared to the control group)

To understand the underlying mechanism by which TgEP induces osteogenic differentiation in hBMSCs, we conducted qRT‒PCR, immunocytochemistry, and western blotting to assess the expression levels of BMP2, Smad1, Smad4, and Smad5 mRNAs, as well as the protein levels of BMP2, Smad1, and p-Smad1/5, which are involved in the BMP/Smad pathway. Following the addition of TgEP to OIM, we observed significant increases in the expression levels of BMP2, Smad1, Smad4 and Smad5 mRNA at 3 days (Fig. 2A). Notably, TgEP treatment also led to elevated BMP2 protein levels at both 3 and 7 days (Fig. 2B, C, D, E). Moreover, TgEP treatment also upregulated the protein levels of Smad1 and p-Smad1/5 (Fig. 2D, E).

**Fig. 3:**
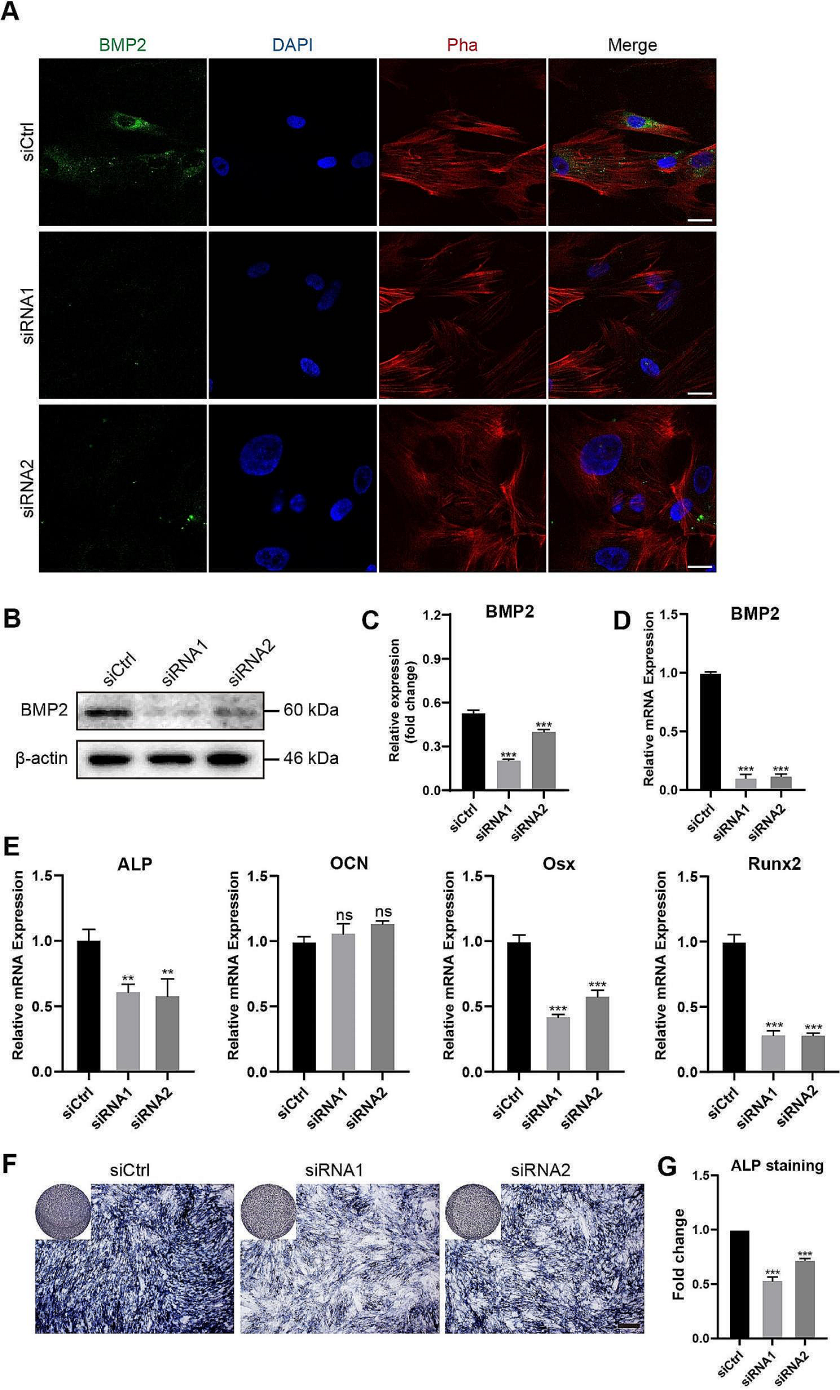
Effect of BMP2-specific siRNA (siBMP2) treatment on the osteogenic differentiation of hBMSCs. (**A**) Immunofluorescence staining of BMP2 protein expression in cells treated with siBMP2. Scale bar = 30 μm. (**B**) Western blotting analysis of BMP2 protein expression in cells treated with siBMP2, with quantification of protein levels normalized to that of β-actin (**C**). (**D**) qRT‒PCR analysis of BMP2 mRNA transcript levels in cells treated with siBMP2. (**E**) qRT‒PCR analysis of the transcript levels of osteogenic target genes in hBMSCs treated with siBMP2. (**F**) ALP staining was used to detect the expression of ALP in cells treated with siBMP2. Scale bar = 500 μm. (**G**) Quantification of ALP staining results using ImageJ software and statistical analysis. (The data are presented as the means ± SDs; *n* = 3; ^**^P < 0.01, ^***^P < 0.001, ^ns^*P*>0.05 compared to the control group)

To determine whether the TgEP-induced osteogenic differentiation of hBMSCs was mediated by BMP2 activation, we performed knockdown experiments using a BMP2-specific siRNA (siBMP2). After siBMP2 treatment for 1 day, the culture medium was replaced with complete MEM-Alpha culture medium, and the cells were cultured for an additional 3 days. To verify the effectiveness of BMP2 knockdown, we performed immunofluorescence staining, western blotting, and qRT‒PCR. The results confirmed the successful knockdown of BMP2 (Fig. 3A, B, C, D). Subsequently, we changed the medium to OIM, and the cells were induced for 3 more days. qRT‒PCR analysis revealed that compared to the control group, the siBMP2 group exhibited significant downregulation of ALP, Runx2, and Osx mRNA transcription, while OCN mRNA transcription was not significantly different (Fig. 3E). Additionally, after 5 days of osteogenic induction, we performed ALP staining to assess the expression of ALP in the cells, and compared with the control group, the siBMP2 group displayed a significant decrease in ALP content (Fig. 3F). These findings suggest that TgEP activates BMP2 and induces downstream Smads proteins, thereby regulating the osteogenic differentiation process via the BMP/Smad signaling pathway.

**Fig. 4:**
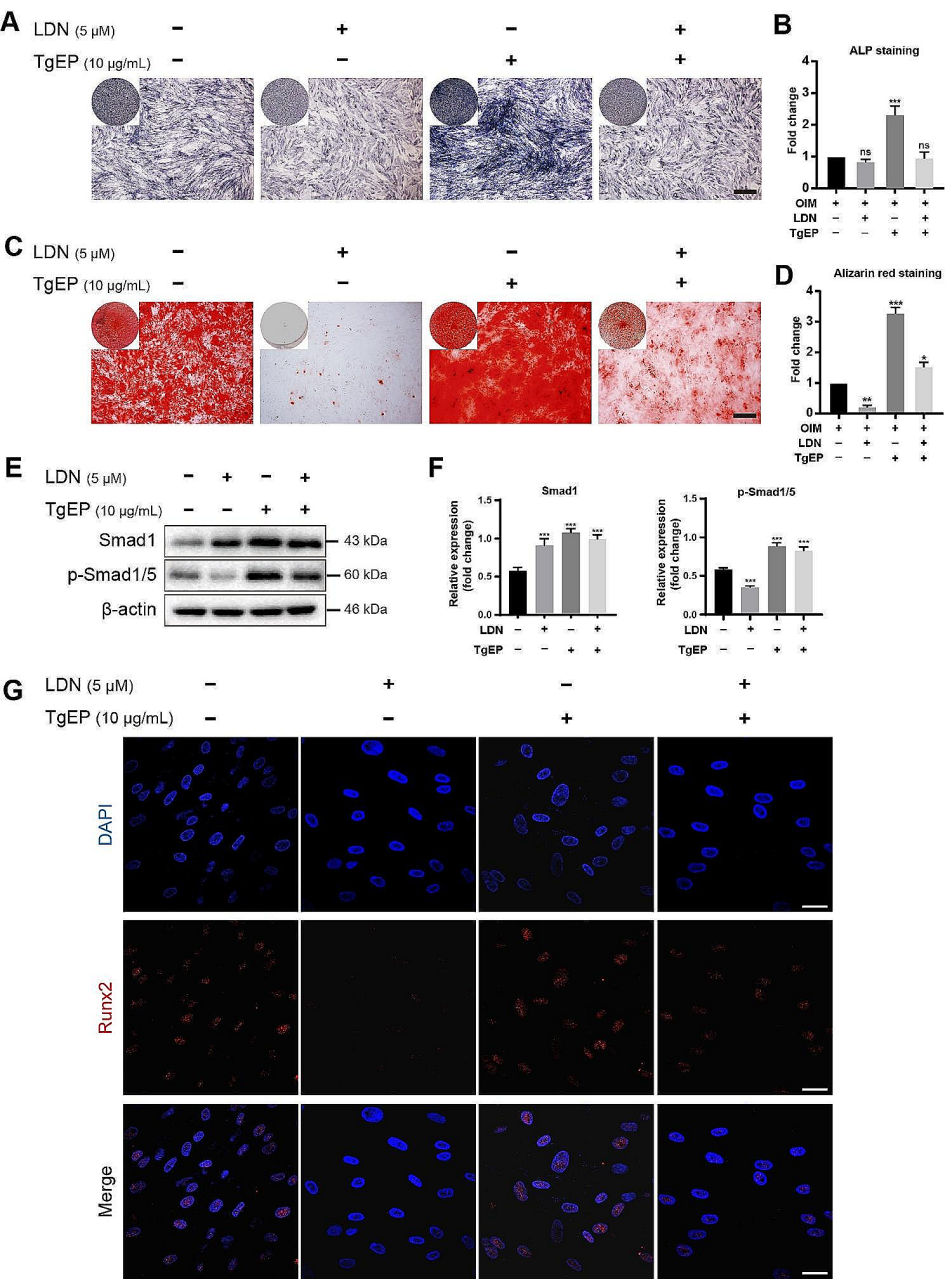
Effect of BMP/Smad pathway inhibitors on osteogenic differentiation of cells. (**A**) ALP staining was used to detect the expression of ALP in cells treated with BMP/Smad pathway inhibitors for 5 days. Scale bar = 500 μm. (**B**) Quantification of ALP staining results using ImageJ software and statistical analysis. (**C**) Alizarin red staining was used to detect the content of mineralized nodules in cells treated with BMP/Smad pathway inhibitors for 7 days. Scale bar = 500 μm. (**D**) Quantification of alizarin red staining results using ImageJ software and statistical analysis. (**E**) Western blotting analysis of Smad1 and p-Smad5 protein expression in cells treated with BMP/Smad pathway inhibitors for 7 days, with quantification of protein levels normalized to that of β-actin (**F**). (**G**) Immunofluorescence staining was used to detect the protein expression of Runx2 in cells; scale bar = 30 μm. (Data are presented as the mean ± SD, *n* = 3, ^*^P < 0.05, ^**^P < 0.01, ^***^P < 0.001, ^ns^*P*>0.05 compared to the control group)

To further validate our findings regarding the role of TgEP in the osteogenic differentiation of hBMSCs, we conducted additional experiments using LDN193189, a specific inhibitor of the BMP/Smad pathway. After 5 days of treatment, we performed ALP staining to assess the expression of ALP in the cells (Fig. 4A, B). After 7 days of treatment, Alizarin Red staining was performed to evaluate the formation of mineralized nodules (Fig. 4C, D). Additionally, after 3 days of osteogenic induction, we conducted immunofluorescence staining to detect the protein expression of Runx2 (Fig. 4G). Importantly, the TgEP-induced increases in ALP expression, mineralized nodule formation, and Runx2 protein expression were significantly inhibited by LDN193189 (Fig. 4A, C, G). Moreover, after 7 days of osteogenic induction, the increase in p-Smad1/5 protein was substantially decreased by LDN193189, while the level of Smad1 protein remained unaffected (Fig. 4E, F). These experimental results further confirmed that the BMP/Smad signaling pathway plays a crucial role in mediating the osteogenic differentiation process triggered by TgEP in hBMSCs.

### The effect of TgEP on osteogenesis in vivo

**Fig. 5:**
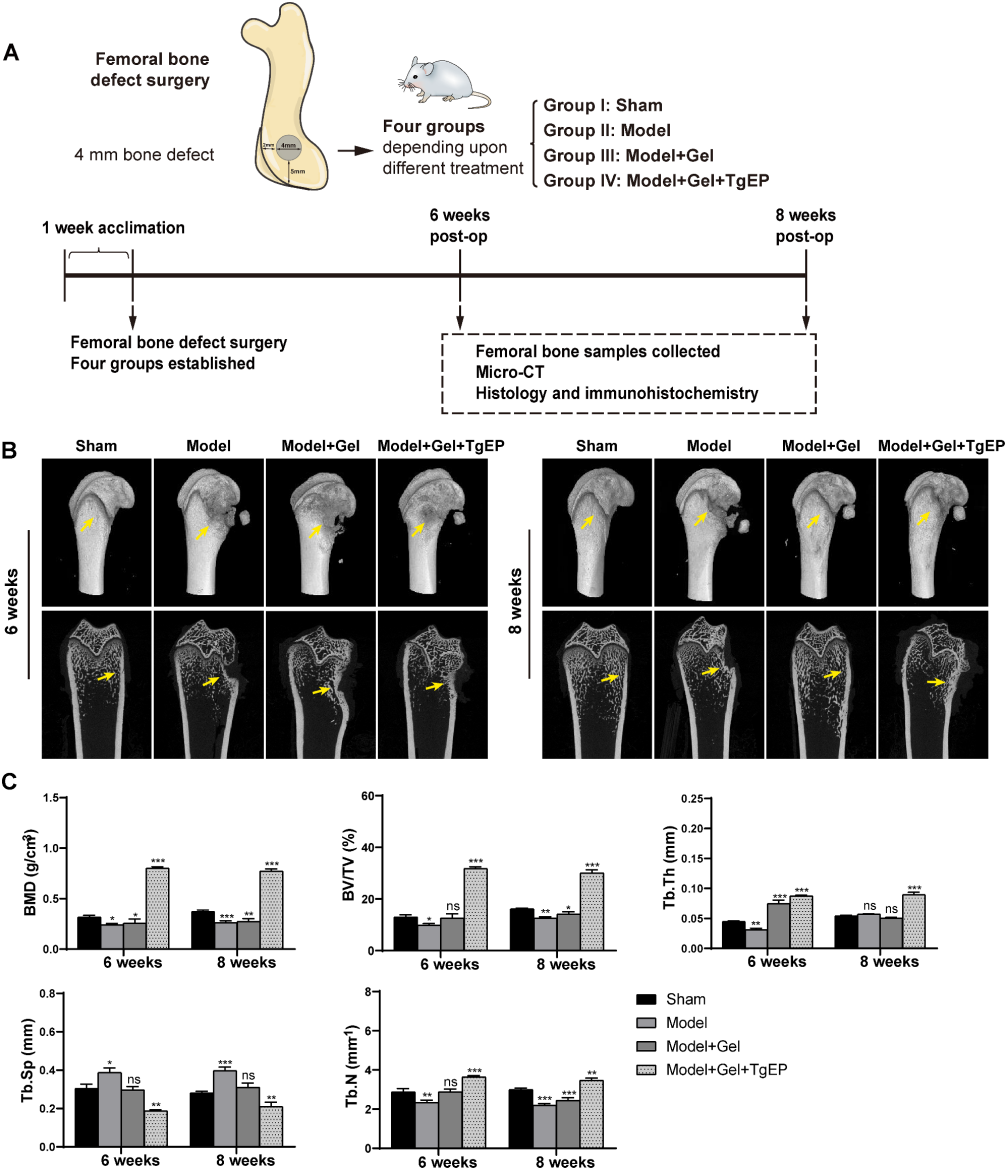
Construction of animal models and the effect of TgEP in vivo. (**A**) Schematic diagram illustrating the experimental procedure for the animal model. (**B**) 3D and 2D images of bone after micro-CT scanning, with yellow arrows indicating the bone defect. (**C**) Statistical analysis of various bone parameters. (The data are presented as the means ± SDs; *n* = 5; ^*^P < 0.05, ^**^P < 0.01, ^***^P < 0.001, ^ns^*P*>0.05 compared with the control group)

To assess the impact of TgEP on bone repair in vivo, a series of animal experiments were conducted (Fig. 5A). The rat femurs in the Model + Gel + TgEP group exhibited superior bone healing compared to those in the Model + Gel group at both 6 and 8 weeks postoperation, as indicated by the 2D and three-dimensional 3D reconstructed images. (Fig. 5B). Compared with those in the Model and Model + Gel groups, BV/TV, BMD, Tb.N, and Tb.Th in the Model + Gel + TgEP group increased (Fig. 5C). The bone parameters showed that the Model + Gel + TgEP group achieved more effective bone repair than the Sham group. This finding suggested that TgEP, in combination with various biologically active substances, has the potential to enhance the bone healing process (Fig. 5C).

**Fig. 6:**
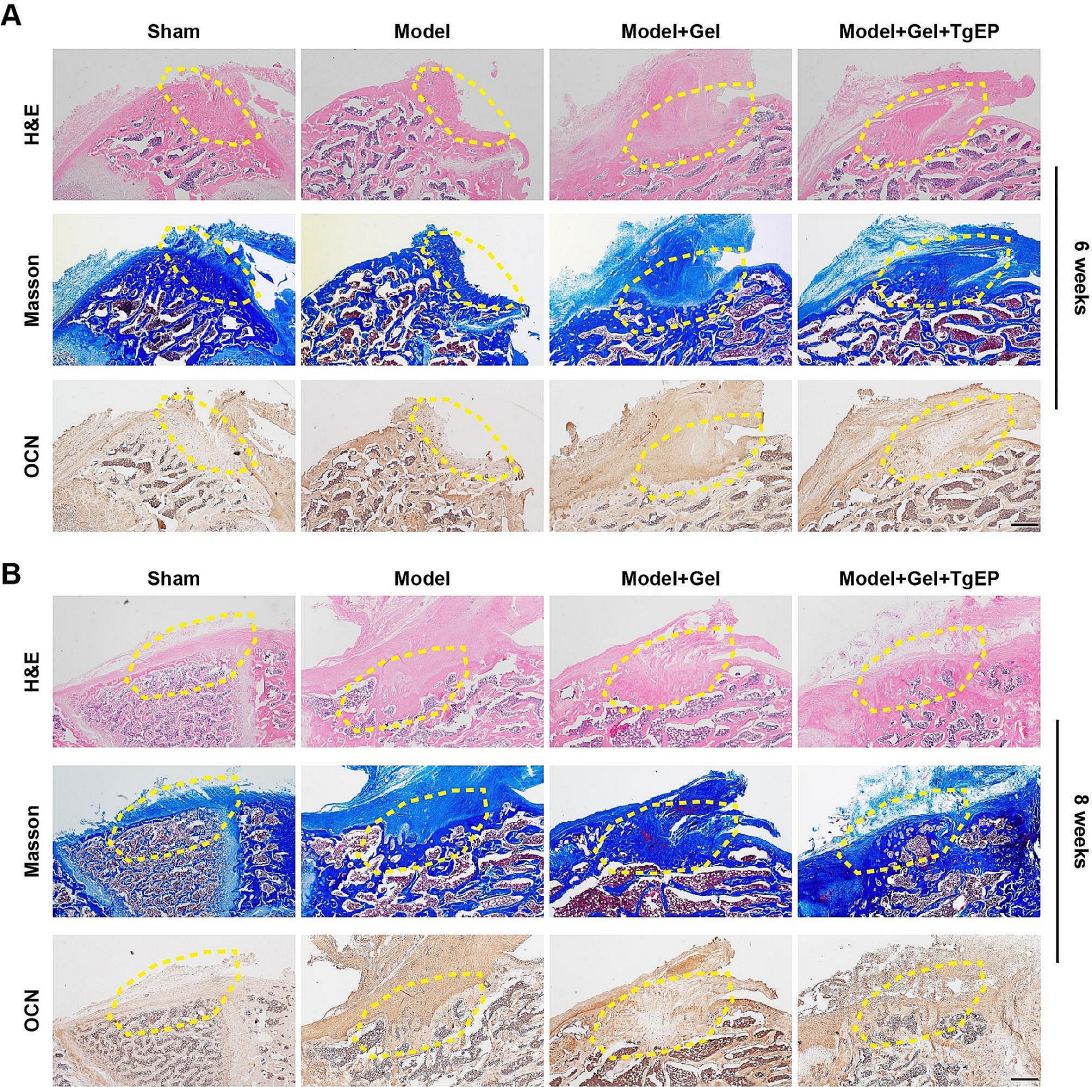
TgEP promotes the repair of bone defects in vivo. (**A**) Histological analysis of bone tissue sections at 6 weeks, including H&E staining, Masson’s trichrome staining, and osteocalcin immunohistochemical staining. Scale bar = 500 μm. (**B**) Histological analysis of bone tissue sections at 8 weeks, including H&E staining, Masson’s trichrome staining, and osteocalcin immunohistochemical staining. Scale bar = 500 μm.

Additionally, histological examinations were performed on femoral samples collected at 6 and 8 weeks postoperation. At 6 weeks, Masson and H&E staining revealed that the defect area in the Model + Gel group was predominantly filled with fibrous tissue and displayed minimal bridging bone formation. In contrast, the Model + Gel + TgEP group exhibited a substantial amount of callus composed of newly formed bone tissue, while the Model group had limited fibrous tissue. OCN histochemical staining revealed a notably increased number of positive cells in the defect area of both the Model + Gel and Model + Gel + TgEP groups, indicating ongoing bone formation. Notably, the Model + Gel + TgEP group exhibited a smaller defect area than the Model + Gel group, highlighting the bone-reforming capacity of TgEP (Fig. 6A). At 8 weeks, Masson and H&E staining showed that the defect area in the Model group was mainly occupied by fibrous tissue and exhibited limited bridging bone formation. In contrast, the Model + Gel group exhibited a dense callus composed of newly formed bone tissue, while the Model + Gel + TgEP group displayed complete cortical bone formation. OCN histochemical staining revealed positive cells in the defect area in all groups except the Sham group. Among them, the Model group exhibited the largest defect area. Conversely, the bone defects in the Model + Gel + TgEP group were fully repaired (Fig. 6B).

## Discussion

The treatment of bone defects has always been a challenging problem in clinical orthopedics, and the process of bone healing relies on osteogenesis, osteoinduction and osteoconduction [40]. In this study, we focused on evaluating the impact of TgEP from *T. gondii* on bone repair, aiming to explore its potential therapeutic applications for promoting bone healing and regeneration. Our findings shed light on the beneficial impact of TgEP on osteogenesis and bone repair.

Our study focused on evaluating the toxicity, proliferation, and osteogenic differentiation of hBMSCs in response to TgEP treatment at the cellular level. Initially, we evaluated the potential toxicity of different concentrations of TgEP on hBMSCs, revealing that concentrations up to 10 µg/mL had no adverse effects on cell viability. Moreover, TgEP at concentrations below 10 µg/mL promoted hBMSC proliferation in a weak but concentration-dependent manner. These findings confirm the safety of the TgEP concentration used in our study. Subsequently, we performed ALP staining, Alizarin red staining, immunofluorescence, qRT‒PCR, and western blotting to assess the osteogenic differentiation of hBMSCs following treatment with various concentrations of TgEP (ranging from 0 to 10 µg/mL). Our findings revealed that TgEP promoted the osteogenic differentiation of hBMSCs in a concentration-dependent manner. Additionally, it is known that *T. gondii* infection in animals can induce the expression of inflammatory cytokines such as IL-1β, IL-10 and IL-23, potentially leading to organ damage [41, 42]. However, our study found no significant differences in the serum concentrations of these cytokines among the experimental groups at any time point (Table SS2-SS4, Fig. SS2), nor did we observe notable changes in the histomorphology of the heart, liver, spleen, lung, and kidney seven days post-surgery (Fig. SS3). These findings indicate that a 10 µg/mL concentration of TgEP at the site of application induces only low levels of animal antigen response and exhibits minimal toxicity, confirming that TgEP’s application in vivo met safety requirements.

The process of bone formation and regeneration involves various factors, including the homing of mesenchymal stem cells, osteoblast formation, and extracellular matrix mineralization [43]. Growth factors play a pivotal role in orchestrating various processes, including cell recruitment, adhesion, proliferation, migration, differentiation, angiogenesis, and other essential functions. Endogenous growth factors include vascular endothelial factor (VEGF), platelet-derived growth factor-BB (PDGF-BB), transforming growth factor-β (TGF-β), bone morphogenetic protein (BMP), and extracellular vesicles [44, 45]. Additionally, exogenous factors such as metformin and traditional Chinese medicine have shown potential in bone regeneration [46, 47]. TgEP, a complex mixture containing proteins such as MIC, GRA, and ROP, exhibits strong immunogenicity and diverse biological functions. These proteins participate in diverse physiological processes in *T. gondii*, including functions such as reproduction, localization, host cell invasion, and regulation of host cell immune responses. In our study, we identified TgEP as a growth factor-like or growth factor-stimulating substance that promotes the osteogenic differentiation of hBMSCs. When incorporated into OIM, TgEP exhibits a synergistic effect, enhancing the osteogenic differentiation process. This finding suggests the potential of TgEP as a valuable factor for augmenting bone regeneration and healing.

Admittedly, the use of TgEP from *T.gondii* in bone regeneration raises legitimate concerns due to its origin from a zoonotic parasite. However, our research demonstrates substantial potential benefits that merit consideration. Our previous studies have shown that during the early stages of osteogenic differentiation, mitochondrial oxidative metabolism in hBMSCs is upregulated while basic glycolysis is downregulated. As osteogenic differentiation progresses, this reduction in glycolysis correlates with decreased osteogenic activity. Intriguingly, treatment with TgEP counteracts these effects by enhancing glycolysis via the Wnt/β-catenin signaling pathway, thus promoting osteogenic differentiation [26]. The utility of substances from sources traditionally considered unsafe for therapeutic purposes is well-documented in medical research. For instance, hirudin, derived from leech saliva, is a highly specific natural thrombin inhibitor used not only to treat thrombotic diseases but also to improve and restore kidney function by modulating inflammatory responses and preventing cellular apoptosis [48]. Similarly, ES-62, a product of filamentous nematodes, has been shown to enhance health and extend lifespan in mice by modulating inflammation, fat metabolism, and gut microbiome dynamics during aging. These examples underscore the potential of parasitic-derived substances, which, despite their origins, have been instrumental in treating diverse ailments [49]. Given the complexity and specificity of the components involved, our future research will focus on isolating and characterizing individual components of TgEP. We will meticulously evaluate their antigenic properties to ensure safety and efficacy in clinical applications. This targeted approach will allow us to harness the beneficial properties of TgEP, mitigating any risks associated with its origin, and ultimately pave the way for innovative treatments in bone regeneration.

**Fig. 7:**
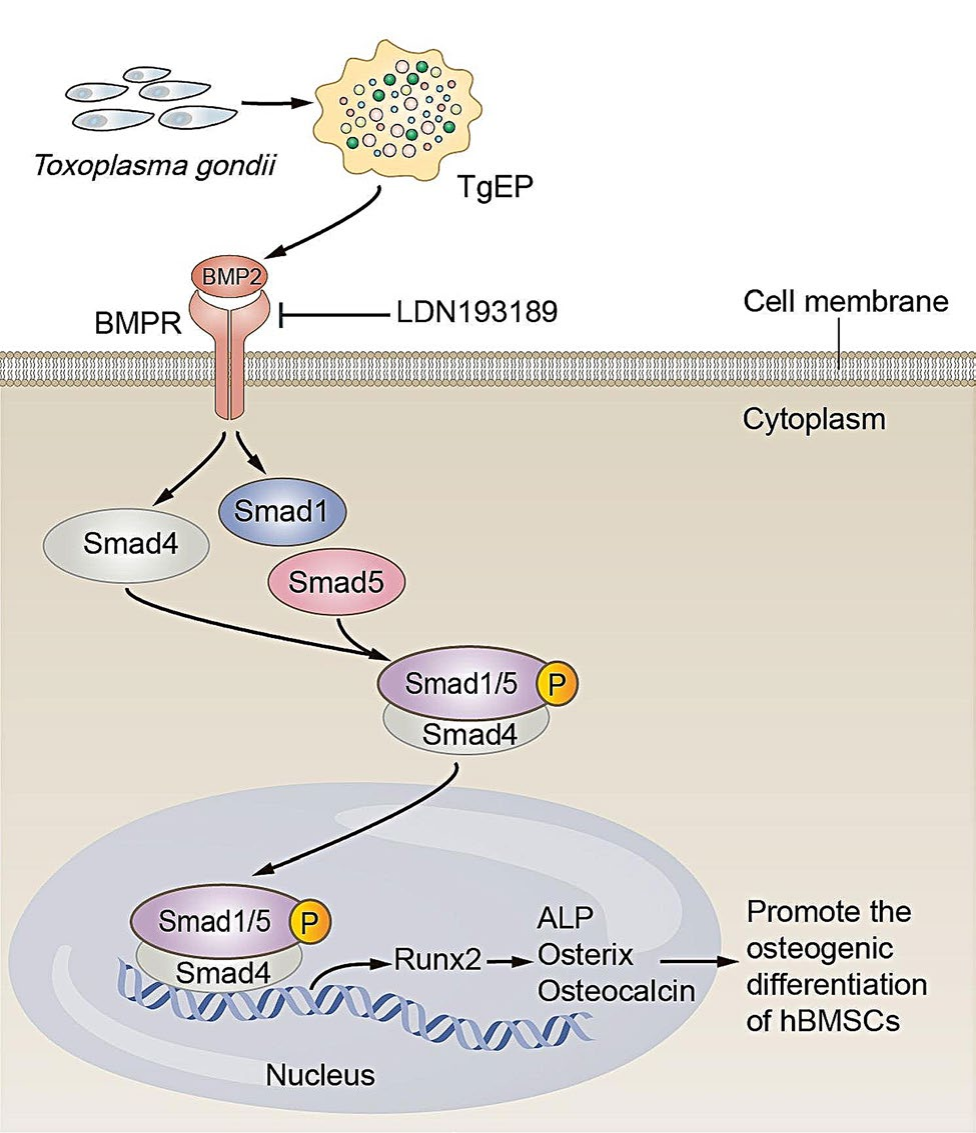
Mechanistic diagram illustrating the regulation of osteogenic differentiation of hBMSCs by TgEP through the BMP/Smad signaling pathway. TgEP enhances the activity of the BMP2 signaling pathway, leading to increased enrichment of p-Smad1/5 and Smad4 complexes through activation of the BMP/Smad signaling pathway. Subsequently, the p-Smad1/5 and Smad4 complexes translocate into the nucleus, where they induce the expression of downstream osteogenic genes

Among the numerous signaling pathways involved in osteogenic differentiation, the BMP/Smad signaling pathway has been the most extensively studied [50]. In this study, we utilized western blotting and immunocytochemistry analyses to investigate the effect of different concentrations of TgEP on protein expression in hBMSCs. Remarkably, at a concentration of 10 µg/mL, TgEP significantly increased the protein expression levels of BMP2, Smad1, and p-Smad1/5. TgEP, a mixture with diverse biological activities, effectively activates the BMP/Smad signaling pathway, a well-known osteogenic signaling pathway, strongly suggesting its significant role in promoting the osteogenesis of hBMSCs. Additionally, the qRT‒PCR results demonstrated a concentration-dependent increase in the transcription levels of BMP2, Smad1, Smad4, and Smad5 mRNA upon treatment with TgEP. To corroborate the involvement of BMP2 in hBMSC osteogenic differentiation and the participation of the downstream signaling molecule Smads in this process, we conducted experiments using BMP2-specific siRNA and BMP/Smad pathway inhibitors (Figs. 3 and 4). The complementary experiments, including the use of BMP2-specific siRNA and BMP/Smad pathway inhibitors, provided further confirmation of the regulatory role of BMP2 and the essential involvement of Smads in the osteogenic differentiation process of hBMSCs. Therefore, we suggest that TgEP promotes osteogenic differentiation by activating the BMP2/Smad signal transduction pathway (Fig. 7). Notably, our findings align with previous research indicating that the promotion of bone formation through the BMP/Smad pathway is correlated with increased expression levels of genes and proteins associated with BMP2, Smads, and other related pathways [51].

Bone tissue engineering is an emerging field that combines principles from engineering and cell biology with the goal of constructing replacement bone in vitro to repair and reconstruct damaged bone tissue [11]. However, this field is still in its early stages of research and lacks an optimal combination of seed cells, cytokines, and scaffold materials for achieving the best outcomes [52]. In our study, we followed the concept of bone tissue engineering by using hBMSCs as seed cells, TgEP as a cytokine-like stimulating factor, and GelMa as the scaffold material. GelMa is an injectable hydrogel and has been widely investigated in various research studies. For instance, Yun Liu et al. demonstrated that GelMA combined with zeolitic imidazolate framework-8 (ZIF-8) could promote extracellular matrix mineralization and effectively treat periodontitis [53]. Furthermore, Zuoying Yuan et al. explored the use of GelMA-based porous shape-memory cryogel microspheres (CMSs) as a cell delivery system to facilitate the formation of bone and blood vessels [54]. The existing studies on GelMa provide valuable evidence supporting its safety and feasibility. In our experiments, the combination of GelMa and TgEP was found to significantly promote hBMSCs osteogenesis in vitro (Fig. S4). With GelMa as the scaffold material, we sought to explore the impact of TgEP on the osteogenic differentiation of hBMSCs and bone repair in vivo. To achieve this goal, we established a bone defect model in SD rats. Our findings highlight the beneficial effects of TgEP on osteogenesis in vivo. The administration of TgEP improved bone repair outcomes, as evidenced by increased bone volume fraction, enhanced bone mineral density, increased trabecular number and thickness, and reduced trabecular separation. The histological analysis further supported these findings, revealing the presence of a thick callus and an increase in bone formation in the presence of TgEP. Collectively, these results reinforce the potential therapeutic applications of TgEP in promoting bone repair and regeneration.

However, it is essential to acknowledge that TgEP is a complex substance with various bioactive components, including hundreds of proteins such as microneme proteins (MICs), dense granule proteins (GRAs) and rhoptry proteins (ROPs) [19]. TgEP is known for its roles in invasion, immune response, and autophagy [55], paralleling the process by which BMSCs differentiate into osteoblasts, a process also linked to autophagy [56]. However, the identification of specific proteins within TgEP responsible for these effects remains incomplete. Consequently, our research investigated the influence of TgEP on the osteogenic differentiation of hBMSCs and its potential mechanisms. The current study, however, does not fully clarify these aspects, which poses challenges to the advancement of clinical bone tissue engineering applications. In the future, it will be imperative to isolate and examine specific protein components within TgEP to better understand their impact on hBMSC osteogenic differentiation and bone repair. Such investigations promise to refine our knowledge of the mechanisms at play and facilitate the development of targeted, well-characterized cell growth factor compositions for bone tissue engineering. This approach will ensure safety, improve treatment monitoring, and enhance traceability. Addressing this knowledge gap will be a priority in our future research endeavors.

## Conclusions

In conclusion, our study revealed that TgEP is a novel stimulator of osteogenic differentiation in hBMSCs, demonstrating its potential for promoting bone repair in vivo. Although further research is necessary to identify the specific components and underlying mechanisms responsible for its effects, our findings provide valuable insights and support the use of TgEP in bone tissue engineering for the treatment of bone defects. The promising results from our study lay the foundation for future investigations aimed at harnessing the therapeutic potential of TgEP in bone regeneration and tissue engineering applications.

### Electronic supplementary material

Below is the link to the electronic supplementary material.

Supplementary Material 1

Supplementary Material 2

Supplementary Material 3

Supplementary Material 4

## Data Availability

The datasets used and/or analyzed during the current study are available from the corresponding author on reasonable request.

## Abbreviations

ALP: Alkaline phosphatase
BMP: Bone morphogenetic protein 2
BMS: Bone marrow mesenchymal stem cells
BMD: Bone mineral density
BV/TV: Bone volume / total volume
OIM: Osteogenic induction medium
Osx: Osterix
OCN: Osteocalcin
TgEP: Toxoplasma gondii excretory proteins
Tb.Th: Trabecular thickness
Tb.N: Trabecular number
Tb.Sp: Trabecular separation
3D: Three-dimensional
