# Supplementary material for: T. gondii excretory proteins promote the osteogenic differentiation of human bone mesenchymal stem cells via the BMP/Smad signaling pathway
Source: J Orthop Surg Res. 2024 Jul 1;19:386. doi: 10.1186/s13018-024-04839-0 (PMC11218376; doi:10.1186/s13018-024-04839-0)
Supplement: Supplementary file 1 — Supplementary Material 1 [file 13018_2024_4839_MOESM1_ESM.docx]

**Table S1** Sequences of primers for qRT-PCR in the present study

| Gene | Primer sequence (5’ to 3’) | | Product length | GenBank accession No. |
| --- | --- | --- | --- | --- |
| *ALP*  *RUNX2*  *Osx*  *OCN*  *BMP2*  *SMAD1*  *SMAD4*  *SMAD5*  *β-ACTIN* | Forward:  Reverse:  Forward:  Reverse:  Forward:  Reverse:  Forward:  Reverse:  Forward:  Reverse:  Forward:  Reverse:  Forward:  Reverse:  Forward:  Reverse:  Forward:  Reverse: | CTTCAAACCGAGATACAAGCACTC  CGTTGTTCCTGTTCAGCTCGTAC  CTCTACTATGGCACTTCGTCAGG  TCAGCGTCAACACCATCATTC  CTCCTGCGACTGCCCTAATT  GTGCGAAGCCTTGCCATACA  GAGGGCAGCGAGGTAGTGAA  TAGACCGGGCCGTAGAAGC  TGTATCGCAGGCACTCAGGTCA  CCACTCGTTTCTGGTAGTTCTTC  TTGGCACAGTCTGTGAACCATGG  GTAACATCCTGGCGGTGGTATTC  CTACCAGCACTGCCAACTTTCC  CCTGATGCTATCTGCAACAGTCC  CAGGAGTTTGCTCAGCTTCTGG  GGTGCTGGTTACATCCTGCCG  CACCATTGGCAATGAGCGGTTC  AGGTCTTTGCGGATGTCCACGT | 137  158  127  182  133  116  106  141  135 | NM_000478.6  NM_001278478.2  NM_001300837.2  NM_199173.6  NM_001200.4  NM_005900.3  NM_001407041.1  NM_001001420.3  NM_001101.5 |

**Fig. S1**


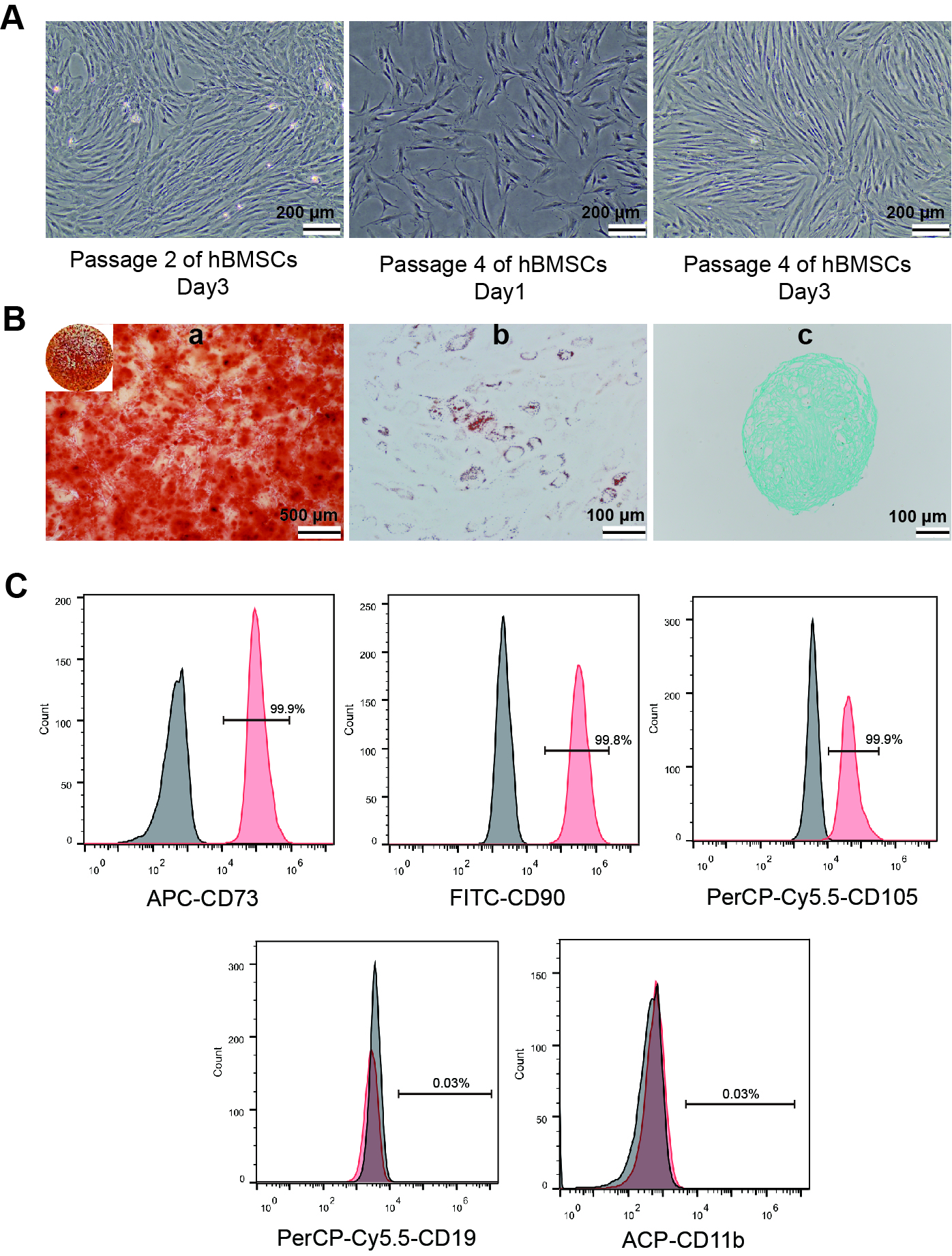


**Fig. S1****.** Identification of human bone marrow mesenchymal stem cells (hBMSCs). (A) Cell morphology of hBMSCs at Passage 2 and Passage 4. (B) Assessment of hBMSCs’ trilineage differentiation ability: Alizarin red staining (a), Oil red O staining (b), and Alcian blue staining (c). (C) Flow cytometry analysis of cell surface markers. The hBMSCs showed positive expression of CD73, CD90, CD105, and negative expression of CD19, CD11b.
