# Supplementary material for: T. gondii excretory proteins promote the osteogenic differentiation of human bone mesenchymal stem cells via the BMP/Smad signaling pathway
Source: J Orthop Surg Res. 2024 Jul 1;19:386. doi: 10.1186/s13018-024-04839-0 (PMC11218376; doi:10.1186/s13018-024-04839-0)
Supplement: Supplementary file 2 — Supplementary Material 2 [file 13018_2024_4839_MOESM2_ESM.docx]

**Table S2** Specific concentrations of IL-1β (pg/mL) in rat serum

| Group | 1 day  pre-op | 1 day  post-op | 7 days  post-op |
| --- | --- | --- | --- |
| Normal | 311.157±25.086 | － | － |
| Model+Gel | － | 564.440±32.201 | 411.210±31.636 |
| Model+Gel+TgEP | － | 575.818±125.419^a^ | 468.337±58.483^a^ |

The data are presented as the means ± SDs; n=5. The normal group exhibited normal IL-1β levels.

^a^*P*＞0.05, compared with the Model + Gel group. (pre-op: before operation, post-op: after operation)

**Fig. S2**

**
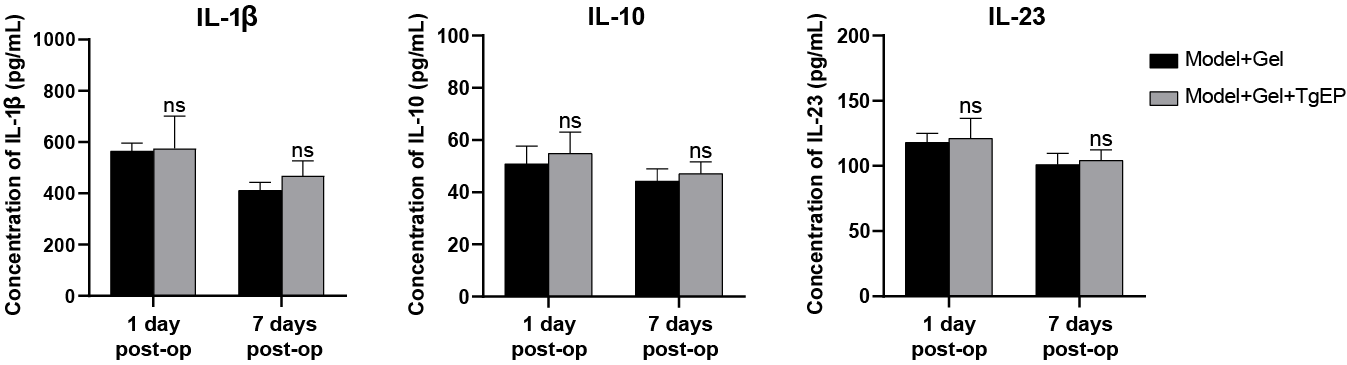
**

**Fig. S2.** Evaluating the antigenicity of TgEP in an *in vivo* rat model. ELISA was used to measure the concentrations of IL-1β, IL-10 and IL-23 in rat serum. (post-op: after operation; data are presented as the mean ± SD, n=5; ^ns^*P*>0.05, compared to the Model + Gel group)
