# Supplementary material for: T. gondii excretory proteins promote the osteogenic differentiation of human bone mesenchymal stem cells via the BMP/Smad signaling pathway
Source: J Orthop Surg Res. 2024 Jul 1;19:386. doi: 10.1186/s13018-024-04839-0 (PMC11218376; doi:10.1186/s13018-024-04839-0)
Supplement: Supplementary file 3 — Supplementary Material 3 [file 13018_2024_4839_MOESM3_ESM.docx]

**Table S3** Specific concentrations of IL-10 (pg/mL) in rat serum

| Group | 1 day  pre-op | 1 day  post-op | 7 days  post-op |
| --- | --- | --- | --- |
| Normal | 37.597±4.302 | － | － |
| Model+Gel | － | 50.875±6.771 | 44.228±4.722 |
| Model+Gel+TgEP | － | 54.863±8.180^a^ | 47.098±4.555^a^ |

The data are presented as the means ± SDs; n=5. The normal group exhibited normal IL-10 levels.

^a^*P*＞0.05, compared with the Model + Gel group. (pre-op: before operation, post-op: after operation)

**Fig. S3**

**
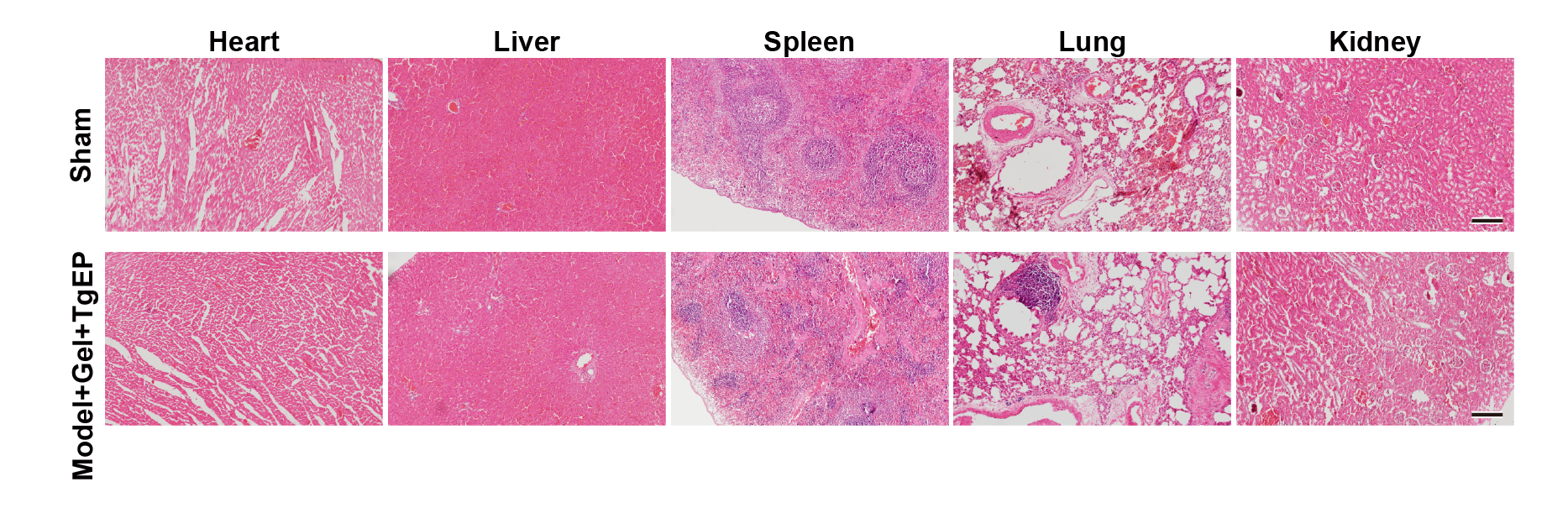
**

**Fig. S3.** Evaluating the antigenicity of TgEP in an *in vivo* rat model. H&E staining of rat heart, liver, spleen, lung and kidney tissue in the Sham group and Model+Gel+TgEP group. Scale bar = 200 μm.
