# Supplementary material for: T. gondii excretory proteins promote the osteogenic differentiation of human bone mesenchymal stem cells via the BMP/Smad signaling pathway
Source: J Orthop Surg Res. 2024 Jul 1;19:386. doi: 10.1186/s13018-024-04839-0 (PMC11218376; doi:10.1186/s13018-024-04839-0)
Supplement: Supplementary file 4 — Supplementary Material 4 [file 13018_2024_4839_MOESM4_ESM.docx]

**Table S4** Specific concentrations of IL-23 (pg/mL) in rat serum

| Group | 1 day  pre-op | 1 day  post-op | 7 days  post-op |
| --- | --- | --- | --- |
| Normal | 96.781±3.607 | － | － |
| Model+Gel | － | 118.115±7.034 | 101.040±8.547 |
| Model+Gel+TgEP | － | 121.144±15.410^a^ | 104.306±8.171^a^ |

The data are presented as the means ± SDs; n=5. The normal group exhibited normal IL-23 levels.

^a^*P*＞0.05, compared with the Model + Gel group. (pre-op: before operation, post-op: after operation)

**Fig. S4**

**
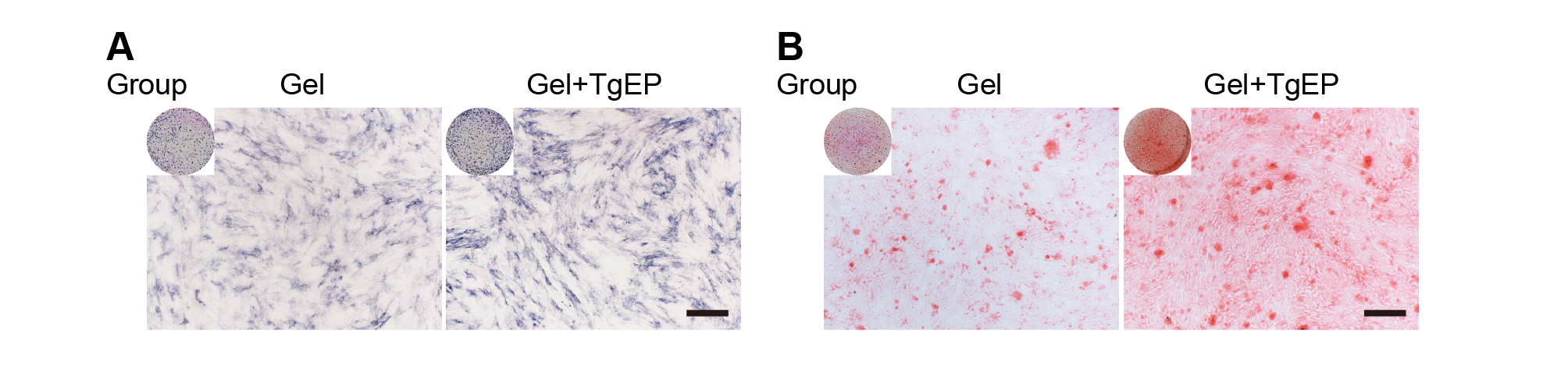
**

**Fig. S4.** Evaluating the osteogenesis of the GelMa hydrogel and TgEP. (A) ALP staining was performed to evaluate ALP expression in cells cocultured with the GelMa hydrogel (Gel) or with the GelMa hydrogel and TgEP (Gel+TgEP). Scale bar = 500 μm. (B) Alizarin red staining was performed to measure the content of mineralized nodules in the cells cocultured with GelMa hydrogel (Gel) or GelMa hydrogel and TgEP (Gel+TgEP). Scale bar = 500 μm.
